#  Etymologia: Creutzfeldt-Jakob Disease

**DOI:** 10.3201/eid2310.171142

**Published:** 2017-10

**Authors:** Douglas John Lanska

**Affiliations:** VA Medical Center, Tomah, Wisconsin, USA; University of Wisconsin, Madison, Wisconsin, USA

**Keywords:** Creutzfeldt-Jakob disease, history of medicine, neuropathology, prions, United States

**To the Editor:** The recent etymologia article on Creutzfeldt-Jakob disease by Henry and Murphy (*1*) does not accurately reflect current understanding of the contributions of Creutzfeldt and Jakob. Although Jakob had reported that Creutzfeldt’s earlier case was a “nosologically very closely connected if not identical affection” (*2*), Creutzfeldt himself later reported that “his case did not bear any resemblance to the cases described by Jakob” (*3*). As discussed by neuropathologist Edgar Peirson Richardson, Jr., in 1977, “Did Creutzfeldt and Jakob describe CJD? … Creutzfeldt probably did not—Jakob to the contrary notwithstanding—and Creutzfeldt is said to have disagreed with the identification of his case with Jakob’s cases. Jakob’s cases, on the other hand, can more readily be fitted into current concepts of the disease without undue strain” (*4*). In 1982, neuropathologist Colin L. Masters and pediatrician D. Carleton Gajdusek concurred with Richardson: “We agree with Richardson (1977) that Creutzfeldt's case probably can be excluded from classification as a spongiform encephalopathy on the basis of his own clinical and pathological descriptions, although a specific alternative diagnosis cannot be made” (*5*). In a later article, Richardson and Masters further noted that Creutzfeldt’s case showed no indication of spongiform change, and the character of the lesions was not characteristic of “CJD” (*6*). In contrast, Jakob clearly described cases of “CJD”: based on reexamination of the original pathologic slides preserved at the University of Hamburg, several of Jakob’s cases were consistent with the clinical picture of “CJD” and showed characteristic pathologic findings of spongiform encephalopathy (*5*). Finally, although Walther Spielmeyer first used the term “Creutzfeldt-Jakob disease” in 1922, his decision to emphasize Creutzfeldt was likely because Creutzfeldt was then working in Spielmeyer’s laboratory; other early terms for the disease gave credit preferentially or solely to Jakob.
